# Supplementary material for: The mitochondrial disulphide relay substrate FAM136A safeguards IMS proteostasis and cellular fitness
Source: Redox Biol. 2025 Oct 8;87:103884. doi: 10.1016/j.redox.2025.103884 (PMC12557607; doi:10.1016/j.redox.2025.103884)
Supplement: Multimedia component 1 [file mmc1.pdf]

SUPPLEMENTAL INFORMATION

The mitochondrial disulphide relay substrate FAM136A safeguards IMS proteostasis and cellular fitness

Christine Zarges[1#], Hanna Fieler[2#], Robin Alexander Rothemann[1], Simon Poepsel[3,4], Lucas T Jae[2,*], Jan Riemer[1,3,*]

1, Redox Metabolism, Institute for Biochemistry, University of Cologne, 50674 Cologne, Germany

2, Gene Center and Department of Biochemistry, Ludwig-Maximilians-Universität München, Munich, Germany.

3, Cologne Excellence Cluster on Cellular Stress Responses in Aging-Associated Diseases (CECAD), University of Cologne, 50931 Cologne, Germany.

4, Center for Molecular Medicine Cologne (CMMC), Faculty of Medicine and University Hospital, University of Cologne, D-50931 Cologne, Germany.

#, equal contribution

* address correspondence to

J.R.: jan.riemer@uni-koeln.de, +49-221-470-7306, ORCID 0000-0002-7574-8457

L.J.: jae@genzentrum.lmu.de, +49-89-2180-71004, ORCID 0000-0002-3531-1760

TABLE OF CONTENT

SUPPLEMENTAL TABLES

Table S1. Cell lines page 3

Table S2. Primers and Plasmids page 4

Table S3. Antibodies page 5

Table S4. Oligonucleotides and siRNAs page 6

Table S5. Further Tools and Equipment page 7

SUPPLEMENTAL FIGURES

Figure S1 page 8

Figure S2 page 9

Figure S3 page 10

Figure S4 page 11

Figure S5 page 12

Figure S6 page 14

SUPPLEMENTAL TABLES

Table S1. Cell lines

Cell line	Plasmid	Gene	Tag (C-terminal)	Reference
**Flp-In T-REx-293**	--	--	--	ThermoFisher R78007
**Flp-In T-REx-293-Mock**	pcDNA5/FRT/TO	--	--	(Murschall et al., 2020)
**Flp-In T-REx-293-FAM136A knockout**	pSpCas9(BB)2A-GFP	Guide: TCCGGAAGATGCAGGTAGCGGGG	--	this study
**Flp-In T-REx-293- FAM136A knockout-Mock**	pcDNA5/FRT/TO	--	--	this study
**Flp-In T-REx-293- FAM136A knockout- FAM136A**	pcDNA5/FRT/TO	ORF *FAM136A*	HA	this study
**Flp-In T-REx-293- FAM136A knockout- FAM136A -9CA**	pcDNA5/FRT/TO	ORF *FAM136A* (T_{103}▶ G, G_{104}▶ C, T_{115}▶ G, G_{116}▶ C, T_{118}▶ G, G_{119}▶ C, T_{157}▶ G, G_{158}▶ C, T_{169}▶ G, G_{170}▶ C, T_{244}▶ G, G_{245}▶ C, T_{256}▶ G, G_{257}▶ C, T_{328}▶ G, G_{329}▶ C, T_{340}▶ G, G_{341}▶ C)	HA	this study
**Flp-In T-REx-293- FAM136A knockout- FAM136A -CA-1**	pcDNA5/FRT/TO	ORF *FAM136A* (T_{103}▶ G, G_{104}▶ C, T_{115}▶ G, G_{116}▶ C, T_{157}▶ G, G_{158}▶ C, T_{169}▶ G, G_{170}▶ C)	HA	this study
**Flp-In T-REx-293- FAM136A knockout- FAM136A -CA-2**	pcDNA5/FRT/TO	ORF *FAM136A* (T_{244}▶ G, G_{245}▶ C, T_{256}▶ G, G_{257}▶ C, T_{328}▶ G, G_{329}▶ C, T_{340}▶ G, G_{341}▶ C)	HA	this study
**Flp-In T-REx-293- FAM136A knockout- FAM136A -Mock**	pcDNA5/FRT/TO and PB-CuO-MCSIRES-GFP-EF1-CymR-Puro	ORF *FAM136A* And --	HA	this study
**Flp-In T-REx-293- FAM136A knockout- FAM136A - FAM136A**	pcDNA5/FRT/TO and PB-CuO-MCSIRES-GFP-EF1-CymR-Puro	ORF *FAM136A* And ORF *FAM136A*	HA and FLAG	this study
**Flp-In T-REx-293-MIA40**	pcDNA5/FRT/TO	ORF *MIA40*	Strep	(Petrungaro et al., 2015)
**Flp-In T-REx-293-MIA40-SPS**	pcDNA5/FRT/TO	ORF *MIA40* (T_{157}▶ G; T_{163}▶ G)	Strep	(Habich et al., 2019)
**Flp-In T-REx-293-MIA40-C53S**	pcDNA5/FRT/TO	ORF *MIA40* (T_{157}▶ G)	Strep	(Fischer et al., 2013)
**Flp-In T-REx-293-MIA40-F68E**	pcDNA5/FRT/TO	ORF *MIA40* (T_{202}▶ G, T_{204}▶ G, T_{203}▶ A)	Strep	(Habich et al., 2019)
**HAP1**	--	--	--	(Carette et al., 2011)
**HAP1 DELE1 knockout**	pL-CRISPR.EFS.tRFP (Addgene 57819)	--	--	(Fessler et al., 2020)
**HAP1 HRI knockout**	pL-CRISPR.EFS.tRFP (Addgene 57819)	--	--	(Fessler et al., 2020)
**HEK293T**	--	--	--	(Habich et al., 2019)
**YME1L knockout**	-	-	-	
**HEK293T-MIA40 knockdown #2**	pSpCas9(BB)2A-GFP	--	--	(Habich et al., 2019)
**HEK293T-MIA40 knockdown #2-Mock**	PB-CuO-MCSIRES-GFP-EF1-CymR-Puro		--	
**HEK293T-MIA40 knockdown #2-MIA40**	PB-CuO-MCSIRES-GFP-EF1-CymR-Puro	ORF *MIA40*	--	
**HEK293T-AIFM1 knockout**	pSpCas9(BB)2A-GFP	--	--	(Salscheider et al., 2022)
**HEK293T-AIFM1 knockout-Mock**	PB-CuO-MCSIRES-GFP-EF1-CymR-Puro		--	(Salscheider et al., 2022)
**HEK293T-AIFM1 knockout-AIFM1**	PB-CuO-MCSIRES-GFP-EF1-CymR-Puro	ORF *AIFM1*	HA	(Salscheider et al., 2022)
**Rosetta2 (DE3)-MIA40**	pGEX-6p-1	ORF MIA40 (C4▶S)	GST (N-terminal)	(Rothemann et al. 2024)

**Rosetta2 (DE3)-MIA40 SPS**	pGEX-6p-1	ORF MIA40 (C4►S; C53►S; C55►S)	GST (N-terminal)	(Rothemann et al. 2024)
**Rosetta2 (DE3)-MIA40 C53S**	pGEX-6p-1	ORF MIA40 (C4►S; C53►S)	GST (N-terminal)	(Rothemann et al. 2024)
**Rosetta2 (DE3)-MIA40 F68E**	pGEX-6p-1	ORF MIA40 (F68►E)	GST (N-terminal)	(Rothemann et al. 2024)

Table S2. Primers and Plasmids

Plasmid	Primer (5´-3´)	Restriction sites
**pMA-T-FAM136A-HA (gene synthesis: gene art)**	--	NheI, KpnI, BamHI, HindIII, BstBI
**pMA-RQ-FAM136A-9CA-HA in (gene synthesis: gene art)**	--	NheI, KpnI, BamHI, HindIII, BstBI
**pMA-RQ-FAM136A-CA-1-HA in (gene synthesis: gene art)**	--	NheI, KpnI, BamHI, HindIII, BstBI
**pMA-RQ-FAM136A-CA-2-HA in (gene synthesis: gene art)**	--	NheI, KpnI, BamHI, HindIII, BstBI
**pGEM4-FAM136A-M28A/L31A/M32A (gene synthesis: GenScript)**	-	KpnI, BamHI
**pGEM4-FAM136A-M28S/L31S/M32S (gene synthesis: GenScript)**	-	KpnI, BamHI
**FAM136A-HA in pCDNA5**	--	KpnI, BamHI
**FAM136A -9CA-HA in pCDNA5**	--	KpnI, BamHI
**FAM136A -CA-1-HA in pCDNA5**	--	KpnI, BamHI
**FAM136A -CA-1-HA in pCDNA5**	--	KpnI, BamHI
**FAM136A-FLAG in PB**	Fw: GCGGCTAGCGGTACCATCTCCATGGCTGAGCTG<br>Rev: CGCTTCGAAAAGCTTGGATCCTTACTTGTCGTCATCGTCTTTGTAGTCGCTTCC	NheI, BstBI
**Fam136A-HA in pGEM4**	--	KpnI, BamHI
**Fam136A-9CA-HA in pGEM4**	--	KpnI, BamHI
**FAM136A guide in pSpcas9(bb)-2a-GFP**	--	Bbs1
**FAM136A guide in pLentiCRISPRv2**	--	Esp3I
**FAM136A guide in pL-CRISPR.EFs.GFP.P2A.Puro**	--	Esp3I

Table S3. Antibodies

Antibody	Company	Identifier
**Rabbit polyclonal anti-FAM136A**	ThermoFisher Scientific	Cat# PA5-56345
**Rabbit polyclonal anti-FAM136A**	Merck	Cat# HPA030104
**Goat anti-Mouse IgG (H&L), HRP Conjugate**	ImmunoReagents	Cat# GtxMu-003-DHRPX
**Goat anti-Rabbit IgG (H&L), HRP Conjugate**	ImmunoReagents	Cat# GtxRb-003-DHRPX
**Mouse monoclonal anti-Actin**	ThermoFisher Scientific	Cat# #MA5-11869
**Rabbit monoclonal anti-ATF4**	Cell Signaling Technology	Cat# #11815
**Mouse monoclonal anti-CHOP**	Cell Signaling Technology	Cat# #2895
**Rabbit polyclonal anti-CLPB**	Sigma-Aldrich	HPA039006
**Mouse monoclonal anti-FLAG**	Sigma-Aldrich	Cat# F3165
**Mouse monoclonal anti-GAPDH**	ProteinTech	Cat# 60004-1-Ig
**Rabbit polyclonal anti-GDF15**	ProteinTech	Cat# 27455-1-AP
**Mouse monoclonal anti-PDH**	Santa-Cruz	Cat# sc-377092; RRID:AB_2716767
**Mouse monoclonal anti-PRDX2**	Sigma-Aldrich	WH0007001M1
**Mouse monoclonal anti-TIM9**	Santa Cruz	Cat# sc-101284
**Mouse Strep-tag antibody**	Qiagen	Cat# 34850
**Rabbit Alexa Fluor 488**	Invitrogen	Cat# A11008
**Rabbit polyclonal anti-AIF**	Merck	Cat# AB16501
**Rabbit polyclonal anti-COX17**	Biorbyt	Cat# orb160552
**Rabbit polyclonal anti-CPOX**	St John's Laboratory	Cat# STJ23214
**Rabbit polyclonal anti-HA**	Sigma-Aldrich	Cat# SAB4300603; RRID:AB_10620829
**Rabbit polyclonal anti-MIA40**	(Erdogan et al., 2018; Fischer et al., 2013)	N/A
**Rabbit polyclonal anti-MIC19**	Proteintech	Cat# 25625-1-AP
**Rabbit polyclonal anti-MIC25**	Proteintech	Cat# 20639-1-AP
**Rabbit polyclonal anti-NDUFA8**	Abcam	Cat# ab184952
**Rabbit polyclonal anti-NDUFS5**	Abcam	Cat# ab179806
**Rabbit polyclonal anti-TOM20**	Santa Cruz	Cat# sc-11415
**Rabbit polyclonal anti-YME1**	Proteintech Europe	Cat# 11510-1-AP
**Rabbit polyclonal anti-HAX1**	St John's Laboratory	Cat# STJ27497

Table S4. Oligonucleotides and siRNAs

Oligonucleotides	Company	Identifier
**FAM136A Guide sgRNA2**<br>Fw: CACCGTCCGGAAGATGCAGGTAGCG	this study	N/A
**FAM136A Guide sgRNA2**<br>Rev: AAACCGCTACCTGCATCTTCCGGAC	this study	N/A
**FAM136A Guide sgRNAex1**<br>**Fw: CACCGACTCCACCGCCTCCTGCACC**<br>**Rev: AAACGGTGCAGGAGGCGGTGGAGTC**	this study	N/A
**FAM136A Guide sgRNAex2**<br>**Fw: CACCGCTGGTGCACCTGCTTCATGG**<br>**Rev: AAACCCATGAAGCAGGTGCACCAGC**	this study	N/A
**Non-targeting control sgRNA**<br>**Fw: CACCGGTATGTCGGGAACCTCTCC**<br>**Rev: AAACGGAGAGGTTCCCGACATACC**	this study	N/A
**DELE1 Guide sgRNA**<br>**Fw: CACCGAGCGACATGTGGCGCCTCCC**<br>**Rev: AAACGGGAGGCGCCACATGTCGCTC**	(Fessler et al., 2020)	N/A
**HRI Guide sgRNA**<br>**Fw: CACCGCCATCGACTTTCCCGCCGA**<br>**Rev: AAACTCGGCGGGAAAGTCGATGGC**	(Fessler et al., 2020)	N/A
**siRNA target sequence**<br>**TGCCAACATCCTGTTTACCAA**	Quiagen<br>SI04228301	N/A
**siRNA target sequence**<br>**TACCAGACTCTTCTTACTACA**	Quiagen<br>SI04331789	N/A

Table S5. Further Tools and Equipment

	Company/ Source	Identifier
**EasyTag™ Express Protein Labeling Mix, [35S]**	Perkin-Elmer	Cat# NEG772
**EasyTag™ Express Protein Labeling Mix, [35S]**	Revvity (Germany) GmbH	NEG772002MC
**ROTI®Quant universal**	Carl Roth	Cat # 0120.1
**TnT Quick Coupled Transcription/Translation System**	Promega	Cat# L1170
**Pierce 660 nm Protein Assay Reagent**	Thermo Scientific	Cat# 22660
**Carbonylcyanid-3-chlorphenylhydrazon (CCCP)**	Sigma-Aldrich	Cat# C2759
**FuGENE HD Transfection Reagent**	Promega	Cat# E2311
**RosettaTM 2 (DE3) SinglesTM Competent Cells**	Novagen	Cat# 70954-3
**One Shot TOP10 Chemically Competent E. coli**	Thermo Fisher	Cat# C404010
**TMRM**	TCI chemicals	Cat# 115532-50-8

Software and Algorithms

**Alpha-fold Multimer**	(Jumper et al., 2021)	
**Pymol**		https://www.pymol.org
**Fiji**	(Schindelin et al., 2012)	https://imagej.net/Fiji
**Image Lab 5.2**	Biorad Laboratories	
**ImageQuant TL 8.1**	GE Healthcare Life Sciences	
**iMTS score**	(Boos et al., 2018)	(http://iomiqsweb1.bio.uni-kl.de/).
**Graphpad Prism**	GraphPad Software, Boston, Massachusetts USA	www.graphpad.com

SUPPLEMENTAL FIGURES

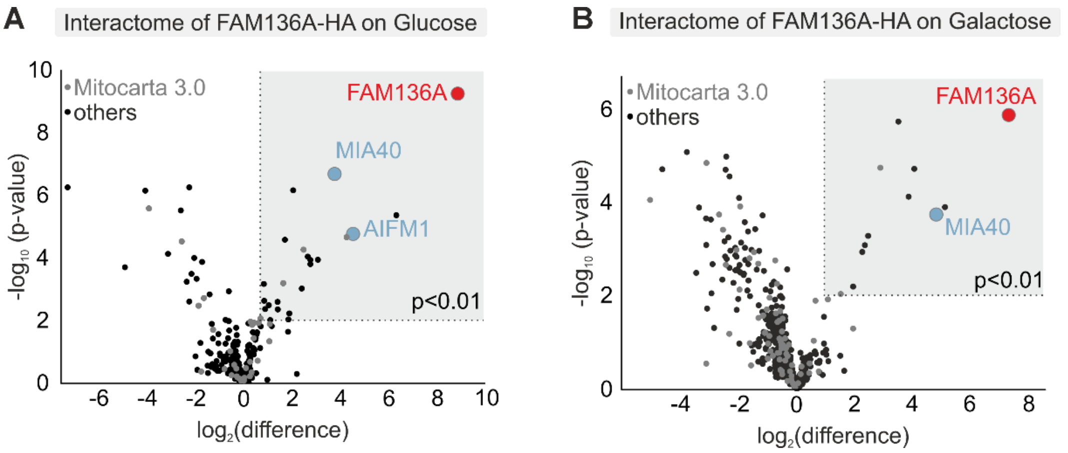

Supplemental Figure S1. FAM136A interacts with MIA40 and AIFM1.

A., B. Interactome of FAM136A-HA from cells grown on glucose (A) or galactose (B) as carbon sources. Cells were treated with NEM to avoid aberrant disulfide formation, and lysed under native conditions. FAM136A-HA was enriched using HA-antibody beads and eluates were analyzed by label-free mass spectrometry. The experiment relies on the analysis of 4 biological replicates. Fold enrichment in the FAM136A-HA IP was plotted for significant hits. N = 4 biological replicates, an unpaired one-sample two-sided Student's t-test was applied ($p<0.01$, log2-enrichment >1).

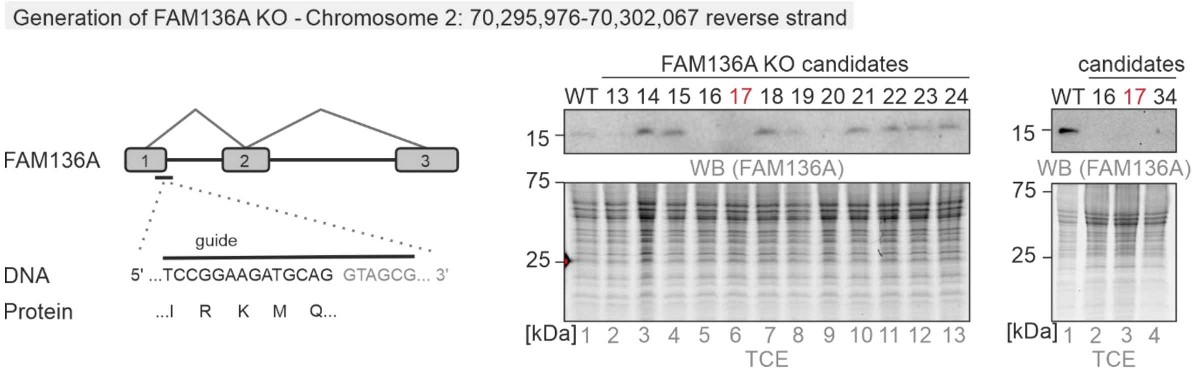

Supplemental Figure S2. Generation of FAM136A knockout cells using CRISPR-Cas technology.

A guide directed against the first exon of FAM136A gave rise to multiple clones. Successful targeting of the gene was confirmed by immunoblotting against FAM136A.

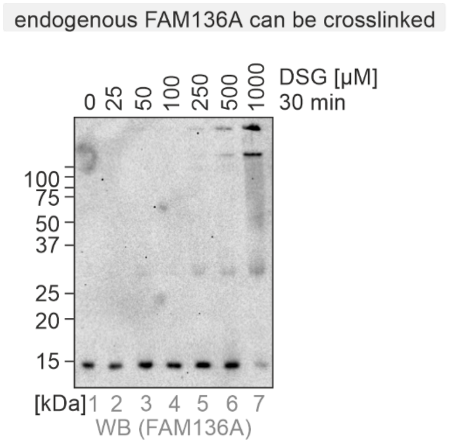

Supplemental Figure S3. Endogenous FAM136A can be crosslinked.

HEK293 cells were incubated for 30 min with the indicated amounts of the crosslinker disuccinimidylglutarat (DSG). The crosslinking reaction was stopped by addition of TRIS. Cells were lysed in loading buffer containing SDS and DTT, and analysed by subsequent immunoblotting against FAM136A. FAM136A can be partially crosslinked to a band of about 30-35 kDa in size, and at higher DSG concentrations also to higher MW oligomers.

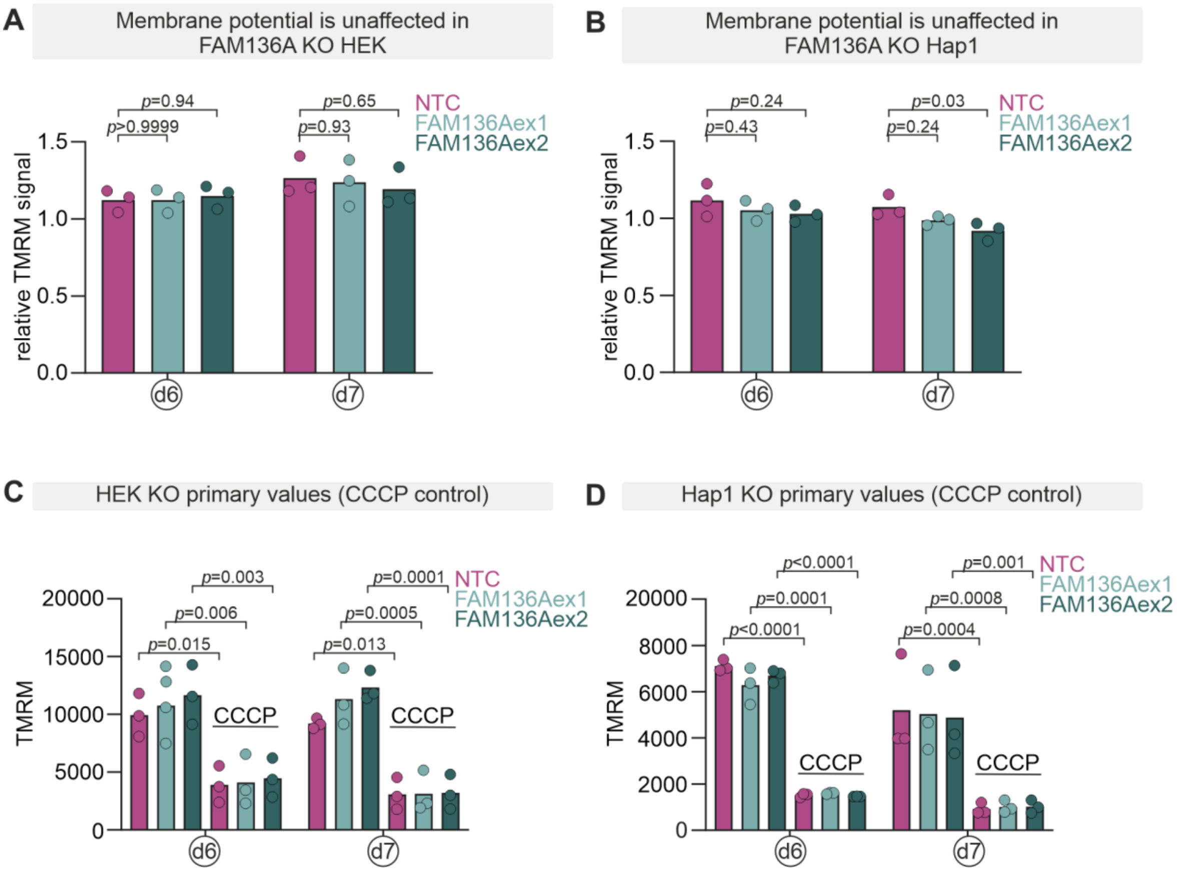

Supplemental Figure S4. Mitochondrial membrane potential upon acute loss of FAM136A.

A., B. HEK293T (A) or HAP1 (B) cells transduced with sgRNAs against FAM136A exon 1, exon 2 or a non-targeting sgNTC were incubated for 30 min with 100 µM TMRM +/- 20 µM CCCP. Cells were measured by flow cytometry and TMRM fluorescence of sgRNA transduced cells was normalised to non-transduced cells from the same well. Two-way ANOVA.

C., D. Primary TMRM values of the transduced cells for comparison with the CCCP positive control. Two-way ANOVA.

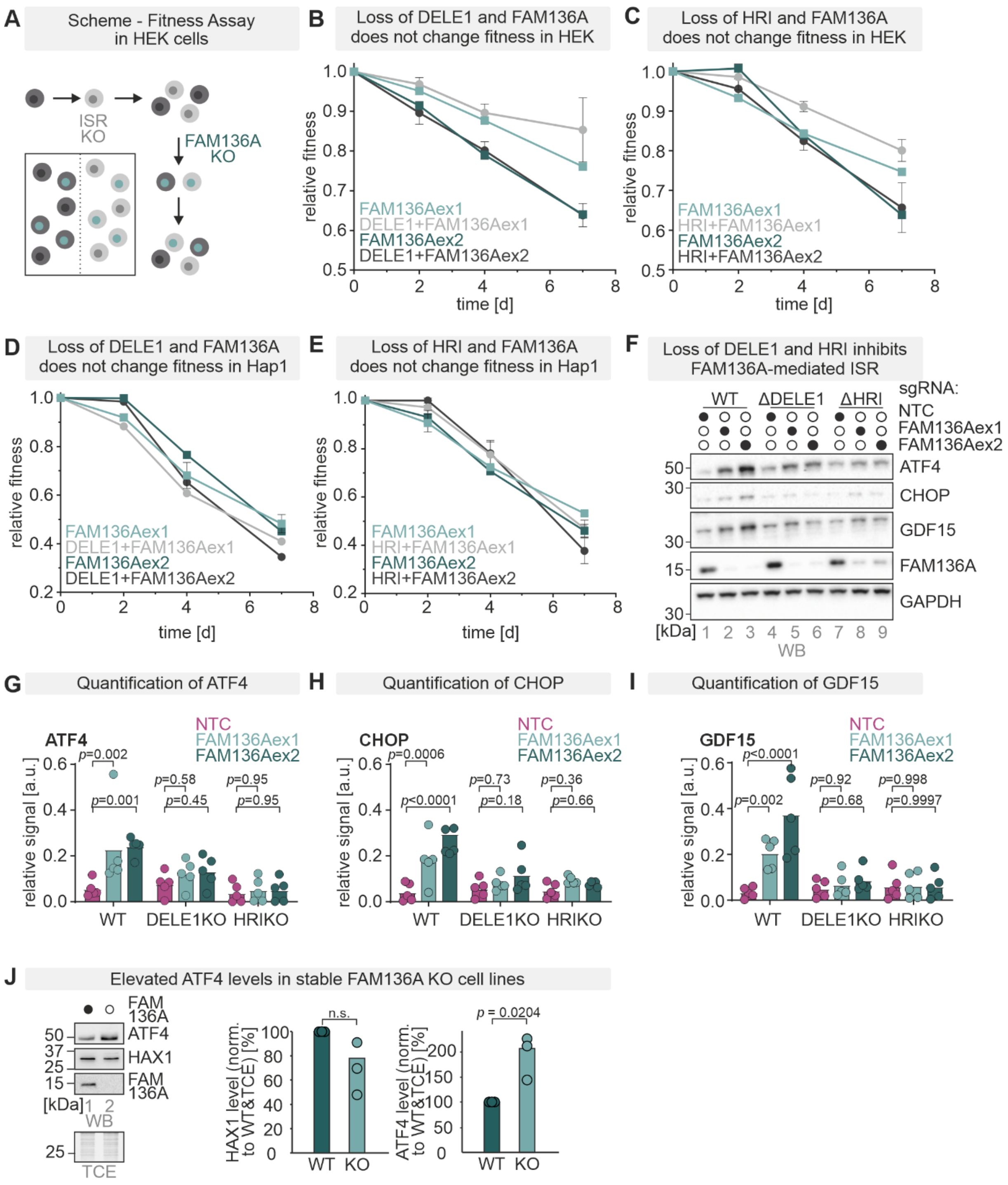

Supplemental Figure S5. The ISR is not responsible for loss of cellular fitness upon FAM136A loss

A.-E. Fitness assay in DELE1/HRI deficient cells. A competitive fitness assay (A) was performed in HEK293T (B., C.) or HAP1 (D., E.) cells. They were transduced with a non-fluorescent non-targeting guide (sgNTC) or GFP-containing guides against DELE1/HRI. sgNTC cells were mixed with sgDELE1/sgHRI respectively and transduced with mScarlet-containing sgNTC or sgRNAs against FAM136A exon 1 or exon 2. The fraction of mScarlet^{+} cells was measured over time and analysed separately for sgNTC or sgDELE1/HRI (GFP^{+}) cells from the same well.

F. HAP1 wildtype or DELE1/HRI deficient cells were transduced with two different sgRNAs to ablate FAM136A. After 6- or 7-days cells were lysed and analysed by immunoblotting for the indicated proteins.

G.-I. Quantification of proteins shown in F. N = 5 replicates. Two-way ANOVA.

J. ATF4 levels are elevated in chronic FAM136A KO cells (n=3) whereas HAX1 levels are not affected by chronic loss of FAM136A (n=3) one-sample t-test.

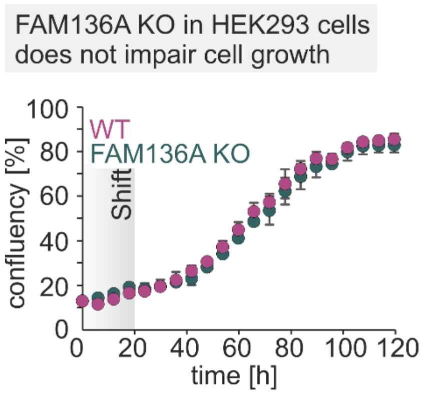

Supplemental Figure S6. Cells with chronic loss of FAM136A adapt

CRISPR/Cas9 FAM136A KO cells do not differ in cell growth from WT cells after exchanging the carbon source after 20h to galactose-containing medium. Cell confluency was measured every 6 hours using Cytosmart OMNI (n=4).
